# Association of serum Ly6/PLAUR domain-containing protein 1 levels with skin sclerosis in systemic sclerosis

**DOI:** 10.1038/s41598-024-56221-z

**Published:** 2024-03-06

**Authors:** Ayaka Sugimori, Issei Omori, Okuto Iwasawa, Hinako Saito, Hibari Nakajima, Ai Matsuno, Shinichi Sato, Hayakazu Sumida

**Affiliations:** 1 Department of Dermatology, Graduate School of Medicine, Faculty of Medicine, The University of Tokyo, 7-3-1, Hongo, Bunkyo-ku, Tokyo, 113-8655 Japan; 2 Scleroderma Center, The University of Tokyo Hospital, Tokyo, Japan; 3 SLE Center, The University of Tokyo Hospital, Tokyo, Japan

**Keywords:** Systemic sclerosis, Biomarkers

## Abstract

Systemic sclerosis (SSc) is a connective tissue disease characterized by aberrant immune activation, vascular injury, and fibrosis of the skin and internal organs. Ly6/PLAUR domain-containing protein 1 (LYPD1) was reported to be secreted and to have various physiological functions such as anti-angiogenic effects. Here we investigated serum LYPD1 levels in SSc patients and the association of serum LYPD1 levels with clinical features of SSc. Serum samples were obtained from 75 SSc patients and 22 healthy individuals as controls. We measured serum LYPD1 levels using enzyme-linked immunosorbent assay kits. Then, the relationship between serum LYPD1 levels and clinical features of SSc was analyzed. Serum LYPD1 levels in diffuse cutaneous SSc (dcSSc) patients were significantly higher than those in the limited cutaneous SSc (lcSSc) patients (median [25–75th percentiles], 1693.43 [1086.61–1917.57] vs. 904.55 [714.356–1285.56] pg/mL), while there were no significant differences in the serum LYPD1 levels between lcSSc and healthy controls (904.55 [714.356–1285.56] vs. 750.71 pg/mL [544.00–912.14]). Further analysis revealed that serum LYPD1 levels in patients correlated with skin thickness scores and serum interleukin (IL)-6 levels, which were known to reflect the extent of skin thickening in SSc. Moreover, serum LYPD1 levels showed a decrease with improvement in skin thickness after treatment, along with a decrease in serum IL-6 levels. These results indicate that LYPD1 might be a potential marker for monitoring skin sclerosis and evaluating the efficacy of skin fibrosis treatment in SSc patients.

## Introduction

Systemic sclerosis (SSc) is an autoimmune idiopathic connective tissue disease, which is characterized by aberrant immune activation, vascular injury, and tissue fibrosis^1^. Microvascular injury and endothelial cell activation are considered the primary events in SSc, which induce fibroblast activation, resulting in skin and certain internal organ fibrosis. Fibrosis of the internal organs can lead to severe complications, such as gastrointestinal complications, interstitial lung disease, pulmonary arterial hypertension, cardiac fibrosis, and scleroderma renal crisis. Digital vasculopathy such as Raynaud’s phenomenon and digital ulcers are also major complications^2–4^. Although advances in SSc treatment have lowered the mortality of SSc, SSc still has higher mortality than any other rheumatic disease^3,5^. Therefore, a better understanding of the disease mechanism and useful biomarkers are needed.

While SSc affects certain organs via inflammation, fibrosis, and vasculopathy, its exact pathophysiology remains elusive. Many cytokines, chemokines, and growth factors have been investigated for their potential involvement in the pathogenesis of SSc. In particular, interleukin (IL)-6 appears to play a key role in inflammation and fibrosis, and its serum levels were reported to reflect skin fibrosis and correlate with modified Rodnan skin score (mRSS) in SSc. Moreover, tocilizumab, a humanized monoclonal antibody against the IL-6 receptor, showed trends for improving skin fibrosis and preventing the progression of lung fibrosis in SSc in randomized controlled clinical trials^6,7^. The mechanism of vasculopathy in SSc is also not fully understood. However, it is known that fibroblasts play an important role in angiogenesis in SSc. For example, it has been proven that pigment epithelium-derived factor and epidermal growth factor-like-domain 7 expressed in fibroblasts have been reported to be involved in SSc vasculopathy^8,9^.

Ly6/PLAUR domain-containing protein 1 (LYPD1), also known as Lynx2, is a member of the Lynx family of neurotransmitter receptor-modulating proteins^10^. LYPD1 is a secreted protein, which was first reported to be expressed in the neurons of the central and peripheral nervous system^11^. It has also been reported to be highly expressed in the fallopian tube and preodontoblasts^12,13^. Although the precise roles of LYPD1 are not fully understood, it has been reported that LYPD1 is an important component of the molecular mechanisms that modulate anxiety, and that LYPD1 mutant mice show increased anxiety-related behavior^14^. LYPD1 is also known to participate in the regulation of ovarian cancer and can act as a novel prognostic marker for ovarian cancer^12^. Furthermore, LYPD1 was reported to be associated with the proliferation and invasion of breast cancer cells^15^. Moreover, it should be noted that LYPD1 was identified as a novel anti-angiogenic factor derived from human heart-derived fibroblasts by suppressing endothelial cell network formation^16^. In this context, GATA6, a zinc finger transcription factor, was recently demonstrated to regulate the anti-angiogenic properties of cardiac fibroblasts through LYPD1^17^.

As mentioned above, LYPD1 has been reported to be expressed in fibroblasts and to be involved in angiogenesis. Given the role of fibroblasts and the contribution of vasculogenesis/angiogenesis in SSc pathogenesis, LYPD1 might be involved in the pathogenesis of SSc. Considering that LYPD1 is a secreted protein, here we tried to measure its level in serum from SSc patients to examine the potential of serum LYPD1 levels as a disease activity marker in SSc.

## Results

### Serum LYPD1 levels were elevated in SSc

**Figure 1:**
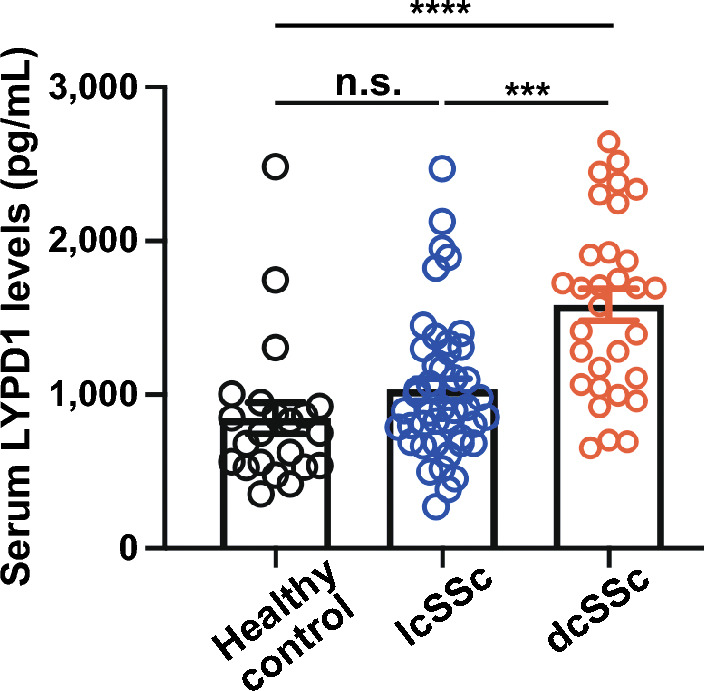
Elevated serum LYPD1 levels in diffuse cutaneous systemic sclerosis patients. Serum Ly6/PLAUR domain-containing protein 1 (LYPD1) levels in diffuse cutaneous systemic sclerosis (dcSSc; *n* = 31), limited cutaneous SSc (lcSSc; *n* = 44), and healthy controls (*n* = 22). ***p < 0.001, ****p < 0.0001.

Serum LYPD1 levels in all enrolled SSc patients were significantly higher than those in healthy controls (median [25-75th percentiles], 1108.00 [820.71–1695.24] vs. 750.71 pg/mL [544.00–912.14], *p* = 0.0004). In the further analysis by the SSc subgroups based on skin involvement^18^, serum LYPD1 levels in dcSSc patients were significantly higher than those in the lcSSc patients (1693.43 [1086.61- 1917.57] vs. 904.55 pg/mL [714.356–1285.56], *p* = 0.0005). On the other hand, there were no significant differences in the serum LYPD1 levels between lcSSc and healthy controls (904.55 [714.356–1285.56] vs. 750.71 pg/mL [544.00–912.14], *p* = 0.2216) (Fig. 1).

### Serum LYPD1 levels correlated with skin scores in SSc

**Figure 2:**
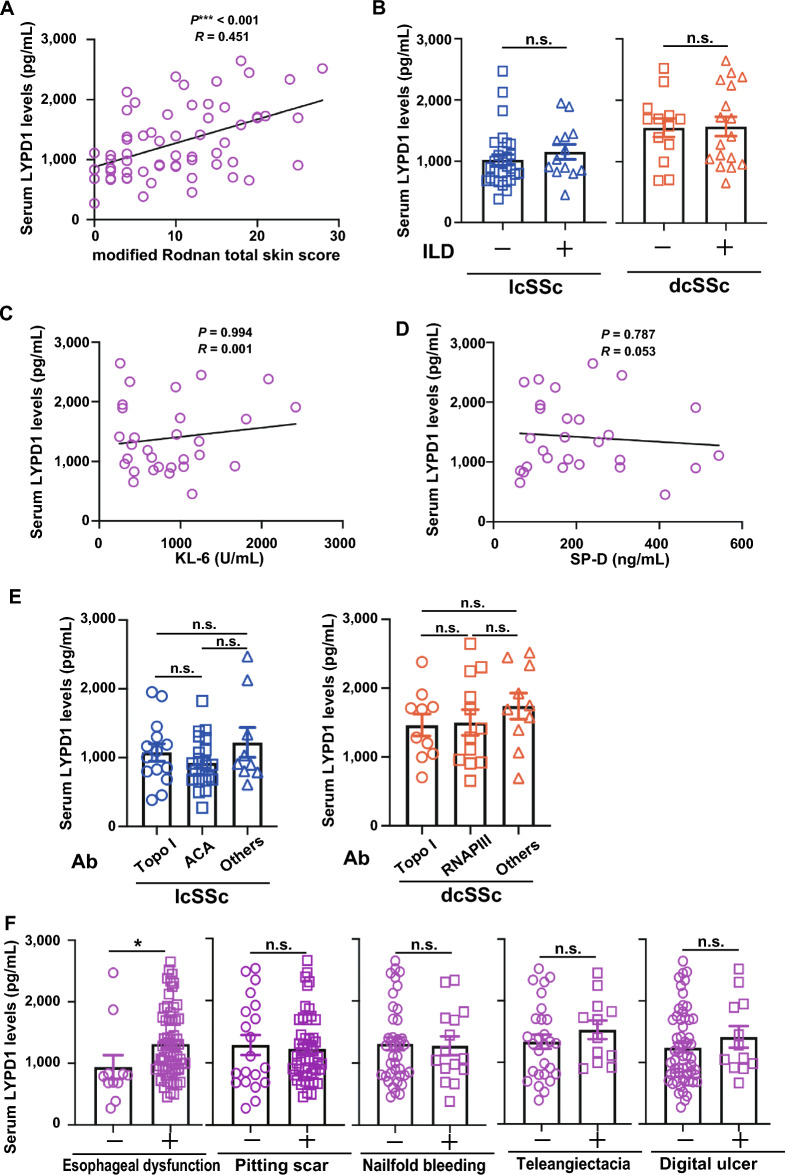
Elevated serum LYPD1 levels were associated with the severity of skin thickness. (**A**) Correlation analysis of serum Ly6/PLAUR domain-containing protein 1 (LYPD1) levels and modified Rodnan total skin thickness score (mRSS) in systemic sclerosis (SSc) patients. The solid line represents the regression line. (**B**) Serum LYPD1 levels of limited cutaneous SSc (lcSSc) patients with or without interstitial lung disease (ILD), diffuse cutaneous SSc (dcSSc) patients with or without ILD. (**C**) Correlation analysis of serum LYPD1 levels and Krebs von den Lungen-6 (KL-6) in SSc patients with ILD. (**D**) Correlation analysis of serum LYPD1 levels and surfactant protein D (SP-D) in SSc patients with ILD. (**E**) Serum LYPD1 levels of lcSSc patients with anti-topoisomerase I (topo I) antibodies, anti-centromere antibodies (ACA) and other antibodies, and those of dcSSc patients with anti-topo I antibodies, RNA polymerase III (RNAPIII) antibodies, and other antibodies. (**F**) Serum LYPD1 levels of SSc patients with or without esophageal dysfunction, pitting scar, nailfold bleeding, telangiectasia, and digital ulcer.

Given that serum LYPD1 levels were elevated in dcSSc but not in lcSSc patients (Fig. 1) and that skin fibrosis is severe in patients with dcSSc, we investigated the relationship between serum LYPD1 levels and the extent of skin fibrosis, which is commonly assessed by mRSS. There was a positive correlation between skin scores and serum LYPD1 levels in SSc patients (*r* = 0.45, *p* = 0.0003) (Fig. 2A). For parameters other than skin thickness, it is known that dcSSc patients have a higher risk of suffering from interstitial lung disease (ILD)^4^. There were no significant differences in serum LYPD1 levels between dcSSc patients with ILD and without ILD (1413.71 [1047.14–2247.12] vs. 1693.43 pg/mL [1282.32–1750.29], *p* = 0.967), nor between lcSSc patients with ILD and without ILD (1032.86 [854.86–1397.70] vs. 896.00 pg/mL [691.43–1164.96] *p* = 0.1942) (Fig. 2B). In support of these results, serum LYPD1 levels showed no correlation with Krebs von den Lungen-6 (KL-6) (*r* = 0.001335, *p* = 0.9944) nor surfactant protein D (SP-D) (*r* = 0.05338, *p* = 0.7873), both of which are well-known serological markers of ILD^19^ including SSc-related ILD (SSc-ILD)^20^ (Fig. 2C,D). In addition, since dcSSc is more often associated with anti-topoisomerase I antibodies and anti-RNA polymerase III antibodies compared to lcSSc^4^, we investigated the association between serum LYPD1 levels and SSc-specific antibodies. However, there were no significant differences in serum LYPD1 levels between dcSSc patients with anti-topoisomerase I antibodies, anti-RNA polymerase III antibodies, and other antibodies (1486.86 [1085.24–1720.98] vs. 1348.02 [948.77–1965.57] vs. 1722.22 pg/mL [1440.57–2233.43], *p* = 0.4751) (Fig. 2E). Similarly, for lcSSc patients, no differences were observed depending on the type of SSc-related antibodies (anti-topoisomerase I antibodies, 1049.25 [806.29–1271.88]; anti-centromere antibodies, 884.86 [683.71–1108.00]; other antibodies, 911.10 pg/mL [806.40–1335.14], *p* = 0.0772) (Fig. 2E).

To explore whether LYPD1 has an association with other fibrotic manifestations, we investigated the association of esophageal dysfunction and serum LYPD1. Patients presenting esophageal dysfunction showed significantly higher levels of serum LYPD1 (740.66 [682.86–823.67] vs. 1188.72 pg/mL [890.43–1693.79] *p* = 0.0374) (Fig. 2F). Since LYPD1 is reported to be involved in angiogenesis^16^, we further examined whether serum LYPD1 levels correlate with clinical features related to SSc vasculopathy. No statistically significant difference can be observed in serum LYPD1 levels between patients with and without nail fold bleeding (1015.14 [691.36–1849.00] vs. 1071.71 pg/mL [857.14–1423.99], *p* = 0.9747) nor between patients with or without pitting scars (1071.71 [906.71–1709.64] vs. 1280.88 [809.20–1700.99], *p* > 0.9999). In addition, there were no significant differences between patients with or without telangiectasias (1176.00 [964.07–1908.54] vs. 1108.00 pg/mL [802.49–1695.24], *p* = 0.2598) nor between patients with or without digital ulcers (1545.93 [1080.27–1844.93] vs. 1303.80 [810.60–1699.38], *p* = 0.2903) (Fig. 2F). These series of results suggest that serum LYPD1 levels are elevated in dcSSc patients, and are particularly correlated to skin fibrosis.

To evaluate the clinical features associated with LYPD1 more specifically, we performed multivariate regression analysis with serum LYPD1 levels as the dependent variable and clinical features including mRSS, esophageal dysfunction, sex, and duration of disease as independent variables. Multiple regression analysis showed that higher mRSS scores were independently associated with higher serum LYPD1 levels (*p* = 0.0013, adjusted R^2^ = 0.1505) (Supplementary Table S1).

### Serum LYPD1 levels correlated with serum IL-6 levels and decreased with improvement in skin thickness after treatment

**Figure 3:**
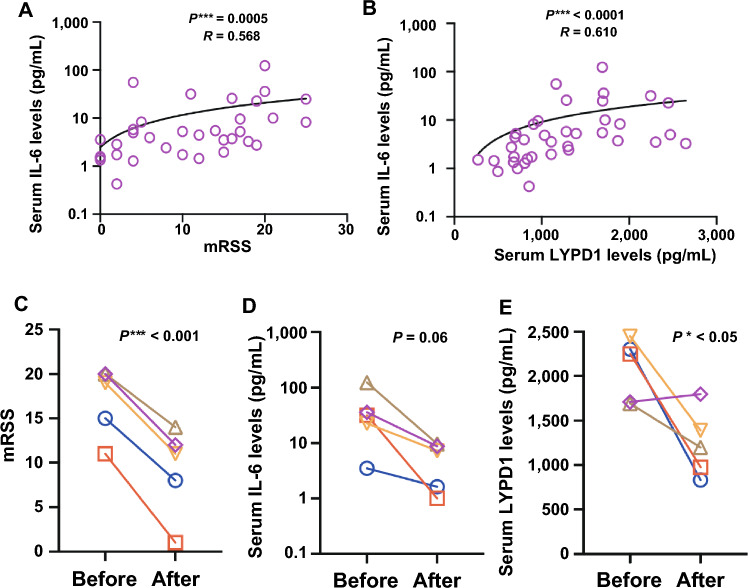
Serum Ly6/PLAUR domain-containing protein 1 (LYPD1) level as a possible marker to monitor the efficacy of skin fibrosis treatment. Correlation of serum IL-6 levels with modified Rodnan skin score (mRSS) and serum LYPD1 levels (**A** and **B**). Changes in mRSS, serum IL-6 levels, and serum LYPD1 levels in five SSc patients before and after receiving immunosuppressive therapies for their skin sclerosis. Plots with the same shape and color from the same patients (**C**–**E**).

Given that serum IL-6 is reported to be elevated in SSc patients and associated with skin scores^21^, we measured IL-6 levels of 34 SSc patients from our cohort, who had enough serum volume left for IL-6 ELISA. There was a significant correlation between mRSS and serum IL-6 levels (*r* = 0.57, *p* = 0.0005) (Fig. 3A), as previously reported^21^. In addition, serum IL-6 levels showed a positive correlation with serum LYPD1 levels (Fig. 3B). Then, based on the correlation between serum LYPD1 levels and skin thickness, we further investigated its clinical usefulness as an indicator of treatment efficacy. In particular, we measured the serum LYPD1 levels of five SSc patients, whose serum samples were collected both before and after treatment for skin sclerosis, to see the changes in serum LYPD1 levels. All of the patients underwent corticosteroid therapy against skin fibrosis, and some of them were also treated with other immunosuppressants such as i.v. cyclophosphamide pulses and mycophenolate mofetil. They showed significant improvement in their skin scores (*p* < 0.0001) (Fig. 3C) with a consistent decreasing trend in serum IL-6 levels (*p* = 0.0625) (Fig. 3D), and their serum LYPD1 levels significantly declined after receiving immunosuppressive therapies (*p* = 0.0416) (Fig. 3E). These results also suggest that serum LYPD1 level could be a useful marker to determine the efficacy of skin fibrosis treatment.

## Discussion

This study revealed that serum LYPD1 levels were significantly higher in dcSSc patients compared to lcSSc patients or healthy controls. Serum LYPD1 levels positively correlated with skin thickness score evaluated by mRSS. Meanwhile, serum LYPD1 levels showed no correlation with the presence of anti-topoisomerase I antibodies in lcSSc patients. Serum LYPD1 levels and the presence of anti-topoisomerase I antibodies or anti-RNA polymerase III antibodies in dcSSc patients had no association. Moreover, there was no correlation between serum LYPD1 levels and the presence of ILD in dcSSc patients nor between serum LYPD1 levels and the presence of ILD in lcSSc patients. These results suggest that LYPD1 may be associated with skin sclerosis rather than the presence of anti-topoisomerase I antibodies, anti-RNA polymerase III antibodies, or the presence of ILD.

Serum LYPD1 levels not only showed a positive correlation with mRSS skin scores in SSc patients but also significantly declined after treatment, along with improved skin sclerosis. These results suggest that LYPD1 might serve as a tool to monitor skin sclerosis. Although mRSS is the most common measure of skin sclerosis in clinical trials and actual clinical practice, its utility has some limitations because of its high interobserver variability^22^. Taking into consideration the result that serum LYPD1 levels decreased with improvement in skin fibrosis after treatment, LYPD1 might be helpful to objectively evaluate the level of skin sclerosis in SSc patients.

A recent study revealed that LYPD1 is an anti-angiogenic factor highly expressed in cardiac fibroblasts^16^. However, in our study, there was no correlation between serum LYPD1 levels and clinical features of SSc caused by vascular damage, such as nail fold bleeding, pitting scars, telangiectasias, or ulcers. Therefore, the correlation of LYPD1 on skin hardening reported here may be due to a new mechanism that is completely different from previous reports. It is unclear whether LYPD1 expressed in fibroblasts is responsible for skin sclerosis or whether LYPD1 expressed in some other cells is involved in skin sclerosis. It has been reported that GATA6 upregulates the expression of LYPD1 in cardiac fibroblasts. Another study suggested that tracheal fibroblast activation by transforming growth factor-β (TGF-β) upregulates the mRNA and protein expression of GATA6^23^. Thus, considering that TGF-β is a critical cytokine in fibrosis and in particular SSc^24^, we speculate that LYPD1 might be downstreamly regulated by TGF-β through GATA6 in SSc pathogenesis.

There are several serum markers previously reported to be correlated with skin fibrosis in SSc such as IL-6, IL-10, B cell-activating factor, IL-33, connective tissue growth factors, TGF-β, soluble tumor necrosis factor alpha (TNF-α)-receptor type 1, CC chemokine ligand 2, CXC chemokine ligand 4, a disintegrin and metalloprotease 12, soluble form of CD93, and soluble inducible costimulator^21,25–33^. Similar to these molecules, LYPD1 is considered to be a secreted protein^11^. To the best of our knowledge, our study is the first to report that serum LYPD1 levels could serve as a biomarker of any kind of disease. LYPD1 might be useful not only in assessing SSc-skin fibrosis but also in fibrosis of other organs or maybe other diseases. Currently, it is unclear whether LYPD1 acts to promote or inhibit fibrosis. In the first place, we have no evidence to support the direct roles of LYPD1 in fibrosis. In addition, the source of LYPD1 in the serum remains unclear. Further studies might be needed to investigate the usefulness and pathological roles of serum LYPD1 levels ​​in various fibrotic pathologies beyond SSc in the future.

This study has several limitations. Our study was a single-center retrospective cohort study and the total sample size was relatively small. As for the analysis of pre- and post-treatment serum samples, further studies with a larger sample size are needed to confirm the present findings. For most of the samples, the serum LYPD1 levels of SSc patients were only measured once before receiving treatment with corticosteroids or other immunosuppressants. Further long-term studies with a larger sample size are necessary to assess whether serum LYPD1 levels are useful in monitoring the degree of skin sclerosis and the effect of treatment targeted to skin sclerosis in SSc. In addition, our study did not include data to reveal the source of increased LYPD1 in the serum. Further studies using patients’ skin samples might be needed to examine that LYPD1 is directly involved in skin hardening.

In summary, this study showed that serum LYPD1 levels were elevated in SSc patients. Moreover, serum LYPD1 levels had a positive correlation with mRSS, while it did not correlate with other clinical features of SSc. Furthermore, to the best of our knowledge, this might be the first study to implicate that LYPD1 is involved in fibrotic diseases. Although further studies are required to clarify the role of LYPD1 in the pathogenesis of SSc skin sclerosis, our study suggests that serum LYPD1 levels may be useful as a serological marker to evaluate the degree of SSc skin sclerosis and monitor therapeutic effects.

## Materials and methods

### Patients

Serum samples, frozen at – 80 °C until assayed, were obtained from 75 SSc patients and 22 healthy individuals as control, who visited the Department of Dermatology at The University of Tokyo Hospital. All patients fulfilled the 2013 ACR/EULAR classification criteria of SSc^34^. Patients were subclassified into: 31 with diffuse cutaneous SSc (dcSSc) and 44 with limited cutaneous SSc (lcSSc) according to LeRoy’s classification system^18^. Patients being treated with corticosteroids or other immunosuppressants at the time of evaluation were excluded from the overall analysis. Patients treated with corticosteroids or other immunosuppressants were included only in the sub-analysis of five SSc patients using pre- and post-treatment serum samples. The therapeutic regimens adopted for each of the five patients at the time of evaluation are as follows. Patient 1: prednisolone (PSL) 20 mg/day, patient 2: PSL 60 mg/day plus intravenous cyclophosphamide, patient 3: PSL 25 mg/day, patient 4: PSL 50 mg/day plus mycophenolate mofetil 1000 mg/day, patient 5: PSL 25 mg/day plus intravenous cyclophosphamide. Written informed consent was obtained from all subjects, and the whole study was performed according to the Declaration of Helsinki and institutional approval (The University of Tokyo, Graduate School of Medicine).

### The measurement of serum LYPD1 and IL-6 levels

Specific enzyme-linked immunosorbent assay (ELISA) kits were used to measure serum LYPD1 levels (MyBioSource, San Diego, CA, USA) and serum IL-6 levels (R&D Systems, Minneapolis, MN, USA) according to the manufacturer’s protocols, as described previously^35,36^. Serum LYPD1 levels and serum IL-6 levels were determined by the accuracy of the curve fit to the standard.

### Clinical assessments

The clinical data of SSc patients were obtained by retrospective review of medical records. Disease duration was defined as the interval between the first clinical manifestation related to SSc other than Raynaud’s phenomenon and the time of blood sampling. The modified Rodnan total skin thickness score (mRSS) was used to evaluate the extent and severity of skin thickness. The details of the assessments are summarized in the legends of Table 1.

**Table 1:** Patient characteristics.

	Healthy controls (n = 22)	dcSSc patients (n = 31)	lcSSc patients (n = 44)
Age, median (IQR) years	46.5 (41.25–57.25)	65 (51–71)	55 (49–68)
Sex, (female/male)	17/5	28/3	42/2
Disease duration, median (IQR) years		1.5 (0.6–2.5)	2.0 (0.5–7.0)
Clinical features			
mRSS, median (IQR)		16 (13.5–19)	4 (2–6)
Raynaud's phenomenon		21 (67.7)	39 (88.6)
Nail fold bleeding		21 (67.7)	30 (68.2)
Pitting scar		5 (16.1)	10 (22.7)
Telangiectasia		7 (22.6)	5 (11.4)
Digital ulcers		4 (12.9)	8 (18.2)
Interstitial lung disease		17 (54.8)	13 (29.5)
Esophageal dysfunction		30 (96.8)	34 (77.3)
Pulmonary hypertension		0 (0.0)	1 (2.3)
Scleroderma renal crisis		1 (3.2)	0 (0.0)
Laboratory findings			
Anti-topoisomerase I		10 (32.3)	14 (31.8)
Anti-centromere		1 (3.2)	21 (47.7)
Anti-RNA polymerase III		12 (38.7)	3 (6.8)

dcSSc, diffuse cutaneous systemic sclerosis; lcSSc, limited cutaneous systemic sclerosis; IQR, interquartile range.

### Statistical analysis

Statistical analysis was performed by Kruskal–Wallis test followed by Dunn’s multiple comparisons test for multiple comparisons. Mann–Whitney *U* test was used to compare the distributions of two unmatched groups, and a paired *t*- or Wilcoxon signed-rank test for the comparison of paired data. Correlations with clinical data were assessed by Spearman’s rank correlation coefficient. Prism (GraphPad, ver. 8) software was used for the above statistical analyses. Multivariate regression analysis was performed with EZR (Saitama Medical Center, Jichi Medical University, Saitama, Japan, ver 4.3.2), which is a graphical user interface for R (the R Foundation for Statistical Computing, Vienna, Austria). A value of *P* < 0.05 was considered statistically significant.

### Supplementary Information

Supplementary Table S1.

## Data Availability

Data are available upon request from the corresponding author.

## References

[1] Varga J, Abraham D (2007). Systemic sclerosis: A prototypic multisystem fibrotic disorder. J. Clin. Investig..

[2] Gabrielli A, Avvedimento EV, Krieg T (2009). Scleroderma. N. Engl. J. Med..

[3] Denton CP, Khanna D (2017). Systemic sclerosis. Lancet.

[4] Allanore Y (2015). Systemic sclerosis. Nat. Rev. Dis. Primers.

[5] Tyndall AJ (2010). Causes and risk factors for death in systemic sclerosis: A study from the EULAR Scleroderma Trials and Research (EUSTAR) database. Ann. Rheum. Dis..

[6] Khanna D (2016). Safety and efficacy of subcutaneous tocilizumab in adults with systemic sclerosis (faSScinate): A phase 2, randomised, controlled trial. Lancet.

[7] Khanna D (2020). Tocilizumab in systemic sclerosis: A randomised, double-blind, placebo-controlled, phase 3 trial. Lancet Respir. Med..

[8] Liakouli V (2018). Scleroderma fibroblasts suppress angiogenesis via TGF-beta/caveolin-1 dependent secretion of pigment epithelium-derived factor. Ann. Rheum. Dis..

[9] Liakouli V (2019). Epidermal growth factor like-domain 7 and miR-126 are abnormally expressed in diffuse systemic sclerosis fibroblasts. Sci. Rep..

[10] Wang N (2019). Long non-coding RNA HULC promotes the development of breast cancer through regulating LYPD1 expression by sponging miR-6754-5p. Onco Targets Ther..

[11] Dessaud E, Salaun D, Gayet O, Chabbert M, de Lapeyriere O (2006). Identification of lynx2, a novel member of the ly-6/neurotoxin superfamily, expressed in neuronal subpopulations during mouse development. Mol. Cell Neurosci..

[12] Sandow JJ (2018). Discovery and validation of novel protein biomarkers in ovarian cancer patient urine. Proteomics Clin. Appl..

[13] Fu Y (2023). Identification of GPI-anchored protein LYPD1 as an essential factor for odontoblast differentiation in tooth development. J. Biol. Chem..

[14] Tekinay AB (2009). A role for LYNX2 in anxiety-related behavior. Proc. Natl. Acad. Sci. USA.

[15] Burnett RM (2015). Organ-specific adaptive signaling pathway activation in metastatic breast cancer cells. Oncotarget.

[16] Masuda S, Matsuura K, Shimizu T (2018). Inhibition of LYPD1 is critical for endothelial network formation in bioengineered tissue with human cardiac fibroblasts. Biomaterials.

[17] Masuda S, Matsuura K, Shimizu T (2023). GATA6 regulates anti-angiogenic properties in human cardiac fibroblasts via modulating LYPD1 expression. Regen. Ther..

[18] LeRoy EC (1988). Scleroderma (systemic sclerosis): Classification, subsets and pathogenesis. J. Rheumatol..

[19] Ohnishi H (2002). Comparative study of KL-6, surfactant protein-A, surfactant protein-D, and monocyte chemoattractant protein-1 as serum markers for interstitial lung diseases. Am. J. Respir. Crit. Care Med..

[20] Sumida H (2018). Prediction of therapeutic response before and during i.v. cyclophosphamide pulse therapy for interstitial lung disease in systemic sclerosis: A longitudinal observational study. J. Dermatol..

[21] Sato S, Hasegawa M, Takehara K (2001). Serum levels of interleukin-6 and interleukin-10 correlate with total skin thickness score in patients with systemic sclerosis. J. Dermatol. Sci..

[22] Merkel PA (2012). Patterns and predictors of change in outcome measures in clinical trials in scleroderma: An individual patient meta-analysis of 629 subjects with diffuse cutaneous systemic sclerosis. Arthritis Rheum..

[23] Li A (2023). GATA6 triggers fibroblast activation and tracheal fibrosis through the Wnt/beta-catenin pathway. Cell Signal..

[24] Ciechomska M, van Laar J, O'Reilly S (2015). Current frontiers in systemic sclerosis pathogenesis. Exp. Dermatol..

[25] Matsushita T (2006). Elevated serum BAFF levels in patients with systemic sclerosis: Enhanced BAFF signaling in systemic sclerosis B lymphocytes. Arthritis Rheum..

[26] Yanaba K, Yoshizaki A, Asano Y, Kadono T, Sato S (2011). Serum IL-33 levels are raised in patients with systemic sclerosis: Association with extent of skin sclerosis and severity of pulmonary fibrosis. Clin. Rheumatol..

[27] Sato S (2000). Serum levels of connective tissue growth factor are elevated in patients with systemic sclerosis: Association with extent of skin sclerosis and severity of pulmonary fibrosis. J. Rheumatol..

[28] Dantas AT (2016). Reassessing the role of the active TGF-beta1 as a biomarker in systemic sclerosis: Association of serum levels with clinical manifestations. Dis. Markers.

[29] Majewski S, Wojas-Pelc A, Malejczyk M, Szymanska E, Jablonska S (1999). Serum levels of soluble TNF alpha receptor type I and the severity of systemic sclerosis. Acta Derm. Venereol..

[30] Hasegawa M (2011). Serum chemokine and cytokine levels as indicators of disease activity in patients with systemic sclerosis. Clin. Rheumatol..

[31] Taniguchi T (2013). Serum levels of ADAM12-S: Possible association with the initiation and progression of dermal fibrosis and interstitial lung disease in patients with systemic sclerosis. J. Eur. Acad. Dermatol. Venereol..

[32] Yanaba K (2012). Augmented production of soluble CD93 in patients with systemic sclerosis and clinical association with severity of skin sclerosis. Br. J. Dermatol..

[33] Yanaba K (2013). Increased production of soluble inducible costimulator in patients with diffuse cutaneous systemic sclerosis. Arch. Dermatol. Res..

[34] van den Hoogen F (2013). 2013 classification criteria for systemic sclerosis: An American College of Rheumatology/European league against rheumatism collaborative initiative. Ann. Rheum. Dis..

[35] Omori I (2023). Serum cold-inducible RNA-binding protein levels as a potential biomarker for systemic sclerosis-associated interstitial lung disease. Sci. Rep..

[36] Urano-Takaoka M (2022). Serum cytokeratin 18 as a metastatic and therapeutic marker for extramammary Paget’s disease. Acta Derm. Venereol..

