# Supplementary material for: Association of serum Ly6/PLAUR domain-containing protein 1 levels with skin sclerosis in systemic sclerosis
Source: Sci Rep. 2024 Mar 6;14:5572. doi: 10.1038/s41598-024-56221-z (PMC10918060; doi:10.1038/s41598-024-56221-z)
Supplement: Supplementary file 1 — Supplementary Table S1. [file 41598_2024_56221_MOESM1_ESM.docx]

**Supplementary Table S1**

Factors associated with serum LYPD1 levels determined by multiple regression analysis.

|  | Estimate | standard error | *p*-value |
| --- | --- | --- | --- |
| Intercept | 791.28 | 186.26 | *p* = 0.0001 |
| mRSS | 33.75 | 10.06 | *p* = 0.0013 |
| Esophageal dysfunction | 199.55 | 196.81 | *p* = 0.3144 |
| Sex | -58.82 | 260.58 | *p* = 0.8221 |
| Disease duration | 2.36 | 12.99 | *p* = 0.8566 |

The multiple regression equation predicting serum LYPD1 levels is as follows.

Serum LYPD1 levels = 791.28 + 33.75 $\times$ mRSS, Adjusted R^2^ = 0.1505

LYPD1, Ly6/PLAUR domain-containing protein 1; mRSS, modified Rodnan skin score
